# Handheld Devices for Food Authentication and Their Applications: A Review

**DOI:** 10.3390/foods10122901

**Published:** 2021-11-23

**Authors:** Judith Müller-Maatsch, Saskia M. van Ruth

**Affiliations:** 1 Wageningen Food Safety Research, Wageningen University and Research, P.O. Box 230, 6700 EV Wageningen, The Netherlands; judith.mueller-maatsch@wur.nl; 2 Food Quality and Design, Wageningen University and Research, P.O. Box 17, 6700 AA Wageningen, The Netherlands

**Keywords:** food fraud, food integrity, on-site detection, portable devices

## Abstract

This review summarises miniaturised technologies, commercially available devices, and device applications for food authentication or measurement of features that could potentially be used for authentication. We first focus on the handheld technologies and their generic characteristics: (1) technology types available, (2) their design and mode of operation, and (3) data handling and output systems. Subsequently, applications are reviewed according to commodity type for products of animal and plant origin. The 150 applications of commercial, handheld devices involve a large variety of technologies, such as various types of spectroscopy, imaging, and sensor arrays. The majority of applications, ~60%, aim at food products of plant origin. The technologies are not specifically aimed at certain commodities or product features, and no single technology can be applied for authentication of all commodities. Nevertheless, many useful applications have been developed for many food commodities. However, the use of these applications in practice is still in its infancy. This is largely because for each single application, new spectral databases need to be built and maintained. Therefore, apart from developing applications, a focus on sharing and re-use of data and calibration transfers is pivotal to remove this bottleneck and to increase the implementation of these technologies in practice.

## 1. Introduction

Over the years, technologies have advanced rapidly and enabled more precise, more efficient, and faster checks of foods for integrity issues to ensure the quality, safety, and authenticity of foods in supply chains. Food authenticity “is about ensuring that food offered for sale or sold is of the nature, substance, and quality expected by the purchaser” [1,2]. Some food fraud cases have impacted on human health, like the addition of melamine to milk powders [3] or the counterfeiting of alcoholic beverages with technical alcohol [4]. Other frauds simply economically disadvantaged consumers, e.g., the passing on of refined olive oil as extra virgin olive oil [5]. When developing new means for food authentication, research has focused mostly on single food commodities, single fraud issues, and/or single technologies. Food authentication methods have traditionally concerned measurement of single markers or a small set of markers with comparison to set thresholds. Over the last two decades, more and more food authentication methods have focussed on analytical signatures, which are based on either known compounds (targeted—profiling) or unidentified features (untargeted—fingerprints) [6,7]. Most of these methods are based on spectrometric or spectroscopic analyses in combination with advanced statistical methods. At the same time, miniaturisation of technologies has allowed more and more use of analytical devices outside laboratory environments [8,9]. This includes noninvasive [10] and handheld, portable devices, some of which are coupled to or even integrated into smartphones [11].

Overviews of these technologies are useful for those that wish to select promising applications or to build further on them. Some studies have reviewed miniaturised applications in this area for particular products. For instance, methodologies/applications focusing on meat [12,13], dairy products [14], and honey [15] as well as plant foodstuffs [16] have been reviewed. These reviews include primarily evaluations of prototypes with a proof-of-concept approach, which are not (yet) operable on a large scale in practice. Despite the reviews mentioned above, technologies for food authentication on the intersection of handheld commercially available technologies and several food commodities have not been reviewed comprehensively till date. 

Therefore, this review aims towards an overview of miniaturised technologies, commercially available devices, and applications for food authentication, or those that at least consider features that are relevant for food authenticity or the identity of foods. Firstly, the available handheld technologies are appraised by their generic characteristics: (1) the type of technologies available, (2) their design and mode of operation, and (3) data handling and output systems. Subsequently, applications of the various technologies are reviewed for food products of animal origin and for those of plant origin. 

## 2. Generic Characteristics of Handheld Devices

### 2.1. Technologies Available in Miniaturised Form

While several technologies that have been used in the past as bench-top instruments to assess food authenticity are being tested in miniaturised form in prototypes, only some have passed the proof-of-concept stage and are available in handheld and lightweight, portable forms. Promising miniaturised technologies include optical sensors, imaging sensors, nuclear magnetic resonance spectroscopy (NMR), and sensor arrays (Table 1).

#### 2.1.1. Optical Sensors

Miniaturised optical sensors that have been applied may scan from ultraviolet (UV, 200 nm) to visible (VIS, 700 nm), and infrared (IR, 2400 nm) wavelengths. Most devices collect the diffuse reflectance or fluorescence spectra arising from interaction of light with solid, paste-like, and even liquid (food) samples. The latter is then called transflectance spectroscopy as the light travels through the liquid sample, reflects at a standardised material, and travels back through the liquid sample to the detector [17,18,20,37]. Depending on this sample constitution and the set of wavelengths used, the penetration depth varies [38]. In contrary to the aforementioned spectrometers, Raman devices collect the spectrum of vibrations upon the interaction of a monochromatic laser light with the sample, and laser-induced breakdown spectroscopy (LIBS) detects the light that emits during the cooling process of plasma produced by the absorption of a laser pulse (Table 1).

#### 2.1.2. Imaging Sensors

Further devices with miniaturised technologies for food screening carry imaging technologies like cameras. For example, prototypes of cameras were used to successfully discriminate horse meat from beef [39] and to assess fruit quality parameters [40,41,42,43,44], fruit bruising [45], or vigor and vegetative expression of vines [46]. The combination of an imaging tool (camera) with technologies like UV, VIS, near-infrared (NIR) as well as Raman spectroscopy is called spectral imaging (Table 1). Thereby, multiple forms are possible, such as hyperspectral imaging (HSI), linescan imaging, and multispectral imaging. HSI collects a three-dimensional hyperspectral data cube that contains not only spectral data but also spatial data. Several prototypes have been described in literature for food fraud testing, e.g., Raman imaging for milk powder authentication [47] or apple contamination [48], short-wave infrared HSI on nut quality [49], or hyperspectral imaging for contamination detection [50,51,52]. Steps to valorise smartphone cameras have been reviewed by Rateni et al., 2017 [11], and McGonigle et al., 2018 [53].

#### 2.1.3. NMR

NMR has been miniaturised to portable and unilateral NMR devices with an open geometry of the respective magnet. Multiple prototypes have been developed and tested in accordance to their food analysis capabilities, ranging from meat and fish to dairy and fruits [30]. Recently, the analysis of packaged foodstuffs [54] and thick samples was researched [55,56]. Nevertheless, commercially available, handheld NMR devices are currently not on the market [30,57], and assessments of food authenticity or food fraud detection in a nontargeted manner are lacking.

#### 2.1.4. Sensor Arrays (Electronic Nose and Tongue)

Whereas the previously mentioned sensors are able to measure a fingerprint of the samples, electronic tongue and nose provide, in addition, selective information on the samples’ ingredients. Both tools intend to mimic the human gustatory and olfactory receptors. Although multiple sensors are available, only a few have been miniaturised. Specific volatiles may be detected by portable electronic noses commonly using conductometric sensors, piezoelectric sensors, or odour-imaging sensor arrays. In contrast, portable electronic tongues detecting solutes in liquids are usually based on amperometry, voltammetry, potentiometry, impedimetry, conductometry, or on a combination thereof (Table 1). There are only a few devices commercially available that may be applied as electronic nose. Several others were tested at prototype stage to assess the authenticity of products of plant origin [58,59].

In the following sections, only devices are included that are (1) available for purchase and are ready-to-use and (2) have been reported in food authenticity assessments in the scientific literature or are aimed at features that could be used for food authentication.

### 2.2. Design and Mode of Operation of Commercially Available Handheld Devices

**Figure 1:**
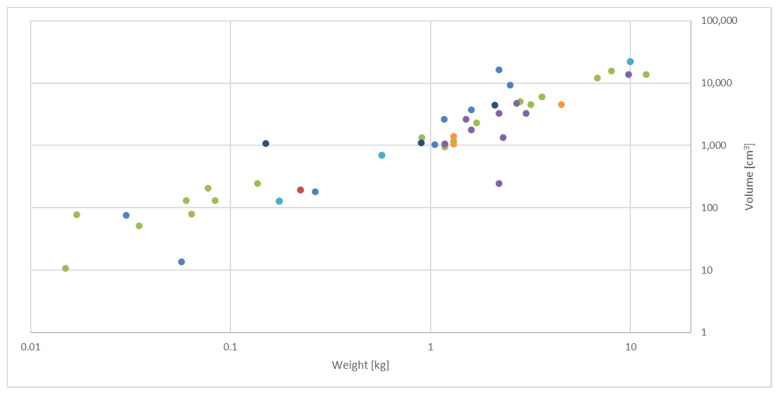
Overview of weight and volume (height × width × length) distribution of some selected commercially available devices used for food authentication according to type of technology (only devices where both weight and size/volume are available are included).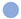 = ultraviolet–visible spectroscopy (*n* = 14); 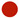 = fluorescence spectroscopy (*n* = 1); 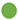 = infrared spectroscopy (*n* = 18); 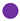 = Raman spectroscopy (*n* = 9); 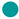 = laser-induced breakdown spectroscopy (*n* = 3); 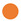 = imaging (*n* = 3); 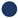 = sensor arrays (*n* = 3). Details of all devices are provided in Supplementary Material—Table S1.

Commercially available handheld devices come in various forms and shapes. Whereas devices carrying optical sensors applying reflectance UV-VIS and IR spectroscopy are available in smaller versions, such as the size of a match box, ones applying laser excitation such as Raman spectroscopy and LIBS are heavier and bulkier. Similar to the latter, imaging devices tend to be larger (Figure 1). Sensor arrays may be implemented in both small and large devices. Although weight and size depend to some extent on the type of technology, some come in large, heavy versions as well as smaller, lighter varieties of devices. Thereby, the size depends on the technology’s parameters used. For example, different IR detectors can run either with or without extra cooling: in small-wavelength ranges, mostly no cooling is required, but additional hardware for cooling is needed when operating at higher wavelengths. Moreover, some instruments are equipped with additional features such as displays or handles, which expand the size and weight of the instruments.

For example, within optical NIR sensors, the lightest commercially available NIR device is the Scio (Consumer Physics, Herzliya, Israel), weighing 0.035 kg and fitting with 6.77 cm × 4.02 cm × 1.88 cm in a hand palm. It is operated via an application on a mobile phone and scans a range from 740 to 1070 nm. In contrast, the LabSpec (Malvern Panalytica, Almelo, the Netherlands (former ASD)) weighs 12 kg and is 12.7 cm × 36.8 cm × 29.2 cm size. The latter NIR device is equipped with a detector for a larger scanning range, 350–2500 nm, and does not need an external operator. 

### 2.3. Data Handling and Output of the Handheld Devices

All handheld devices have in common that a database with reference values and/or calibration curves is necessary to ensure the foods’ integrity [60]. While a database containing food fingerprints is usually custom-made, e.g., for geographical origin determinations of ham or for the grade of olive oil, some databases contain spectra of common adulterants or contaminants in various concentrations for spectral matching. The databases are tailormade for each device, and only lately approaches and algorithms have been developed to make databases/reference values applicable for multiples devices [7]. Some companies have open-access databases that are populated by users, such as, for example, Scio (Consumer Physics, Herzliya, Israel). Other companies offer the possibility to purchase tailormade databases or support creation of one by the applicant, such as all devices of ThermoFisher Scientific (Waltham, MA, USA).

Often pretreatment needs to be applied to spectroscopic data to convert the spectra from reflectance to absorbance values, reduce unwanted light-scattering effects, and to eliminate baseline shifts and background information (noise) in the data. In case of spectral imaging, the 3D data cube needs to be transferred into 2D and the spectral data treated consecutively. Depending on the aim, three different kinds of approaches in modelling may be applied: (1) classifying or discriminating normal from unusual, which is called “broad anomaly testing”, (2) classifying or discriminating different (multiple) groups, e.g., species, varieties, and geographical origins, and (3) measuring the degree of adulteration in case there is a limit of adulterant that is still legal. Commonly used chemometrics for the pretreatment of spectroscopic data, the construction of models based on it, and their transfer between instruments and food commodities have been addressed by Capitan-Vallvey and Palma, 2011 [61], Oliveira et al., 2019 [6], and McGrath et al., 2018 [7]. Similar approaches are used in the application of electronic noses and tongues as recently summarized and reported by [31,33,37].

## 3. Current Applications of Handheld Devices for Food Authentication

The current applications in food authentication with commercial, handheld devices target products of animal origin as well as plant origin. In the following sections, applications and their performance indicators (classification rates, accuracy, etc.) are reviewed according to food commodity. For each commodity group, applications are presented in order of applied technologies: VIS, fluorescence spectroscopy, NIR, Fourier-transformed IR (FT-IR), mid-IR (MIR), Raman spectroscopy, LIBS, imaging, and sensor arrays.

### 3.1. Food Products of Animal Origin

#### 3.1.1. Meat, Meat Products, and Offal

In the scientific literature on the authentication of meat, meat products, and offal, only one handheld device using solely VIS wavelengths from 400 to 700 nm was reported. Dian et al., 2008 [62], classified the feeding regime of, respectively, 91% and 99% of pasture-fed and stall-fed lambs correctly. In spite of the former study, most applications included multiple wavelength ranges or technologies, such as VIS-NIR, NIR, Raman, LIBS, and sensor array technology (Supplementary Material—Table S2). 

With VIS-NIR devices, indirect authentication of pig carcasses according to feeding regime has been proven successful. Perez-Marin et al., 2009 [63], predicted fatty acid concentrations in the transverse section of pig carcasses, in particular, linoleic acid (R^2^_CAL_ 0.64), oleic acid (R^2^_CAL_ 0.90), stearic acid (R^2^_CAL_ 0.84), and palmitic acid (R^2^_CAL_ 0.93). These fatty acids were subsequently used to assess the feeding regimes. Similarly, Prieto et al., 2018 [64], showed that the ratio between polyunsaturated and saturated fatty acids (R^2^_P_ value of 0.93 and Root mean square error of cross-validation (RMSEP) of 0.019%) and iodine value (R^2^_P_ 0.94 and RMSEP 1.03%) could be predicted reasonably well. Prieto et al., 2015 [65], detected the unapproved postmortem moisture enhancement of pork with salt and di-sodiumphosphate solution using a wavelength range of 350 to 2500 nm. Non-moisture-enhanced and moisture-enhanced samples, respectively, were correctly identified after two days of aging with a success rate of 99% and 94%, respectively. After 14 days of aging, 94% of the same non-moisture-enhanced samples and 92% of the moisture-enhanced samples could still be correctly classified. Dixit et al., 2020 [66], evaluated two sensors, a VIS-NIR (350–2500 nm) and a NIR device (900–1700 nm), to determine the age of slaughtered lambs. The authors concluded that both sensors were able to differentiate two age groups, i.e., the early (4 months) and late-season (12 months) lambs.

In addition, NIR devices using solely wavelengths above 700 nm (Supplementary Material—Table S2) showed promising results when applied for the authentication of meat, meat products, and edible offal. To authenticate the correct premium classifications based on feeding regimes of pigs, different NIR devices were tested. Using a wavelength range from 900 to 1700 nm, 93% [67] to 99% [68] of all samples were identified correctly. In contrast, Zamora-Rojas et al., 2012 [69], achieved a correct classification rate of 61–96% for pork carcasses. Horcada et al., 2020 [70], reported a 76% correct classification rate when distinguishing free-range pork carcasses, carcasses from animals fed on acorns and grass with supplements, and ones from animals fed on compound feeds with the same instrument. Moreover, a high correct classification rate was determined for chicken meat quality classifications based on feeding regime. Over 95% were correctly identified according to standard feeding, free-range, or corn-fed feeding as well as the feeding regimes according to the Dutch animal welfare classification system ranging from conventional to organic [71]. Great classification predictions were reported when discriminating chicken parts (99% correct) when using a wavelength range of 900–1700 nm [72] or when discriminating different species. Dumalisile et al., 2020 [73], applied a range between 908 and 1700 nm to classify different game species and achieved 81%, 92%, and 97% correct classification for impala, ostrich, and eland muscles, respectively. When mixing veal sausages with pork or pork fat, a 100% correct discrimination rate above a 10% (*w*/*w*) adulteration level was established by Schmutzler et al., 2015 [74]. Similar discrimination results were obtained in ground meat blends, e.g., in binary blends of chicken/beef (R^2^_p_ 0.99, RMSEP 3.5% (*w*/*w*)) and ternary blends of beef/chicken/pork (R^2^_p_ 0.93, RMSEP 4.7% (*w*/*w*)) by Silva et al., 2020 [75].

To identify substitution of meat products with meat from other species or offal, portable LIBS sensors have been evaluated as well. Bilge et al., 2016 [76], evaluated minced beef blended with pork or chicken in a ratio of 10–50% (*w*/*w*). Using the five-channel Aurora LIBS spectrometer, determination coefficients (R^2^) and limits of detection (LOD) of 0.994 and 4.4% for pork adulteration and 0.999 and 2.0% for chicken adulteration, respectively, were reported. Less successful but still worth mentioning was the detection of minced beef substituted with offal (liver) indirectly via copper concentrations with a R^2^_p_ of 0.85 and an RMSEP of 37 ppm by Casado-Gavalda et al., 2017 [77].

In brief, the macro composition of meat products may be detected sufficiently using NIR technology, which allows the detection of fraudulently added moisture to meat products. By adding the VIS wavelength results, greater precise is achieved and maybe also detailing of minor components, such as fatty acid concentrations, allowing further details of authenticity traits such as species detection or production system. Hence, classifications on organic or nonorganic, feeding style, as well as breed might be conducted successfully using NIR devices or VIS-NIR devices. The detection of meat substitution by meat from other species or offal may be detected using both NIR and LIBS devices. Despite the above, there is still limited information about the abilities of VIS, LIBS, Raman spectroscopy, and fluorescence spectroscopy, as well as spectral imaging and sensor array applications for meat authentication. 

#### 3.1.2. Milk and Milk Products

Applications for the authentication of milk and milk products are listed in Supplementary Material—Table S3. The application of VIS devices and ones applying fluorescence spectroscopy for the authentication of milk and milk products is limited. One device was applied to assess the feeding regime of cows in cheeses derived from the cows’ milks using a wavelength range between 400 and 700 nm. In this case, 79% to 91% of the cheeses were classified correctly, which is considerably lower than the 96% correct classification achieved using a benchtop NIR device in the same study [78]. 

Several scientific reports evaluated the ability to assess authenticity according to fat, protein, and carbohydrate content (especially lactose) of milk and milk products with NIR. Uusitalo et al., 2019 [79], compared three portable NIR sensors, covering wavelength ranges of 1100–1400 nm, 1700–2000 nm, and 2200–2500 nm in regard to their ability to assess fat, protein, and lactose contents of raw milk, drawing subsequent conclusions on the feeding regime of the cows. The authors concluded that the prediction was successful, however not sufficient for the application by legal entities. Using the VIS wavelength range and parts of the shortwave NIR range (400–995 nm), Bogomolov et al., 2017 [80], were able to sufficiently predict fat content (R^2^_CV_ 0.975 RMSECV 0.090%) and protein content (R^2^_CV_ 0.84 RMSECV 0.079%) in retail milk, which was used to authenticate genuine milk. Despite the low success rates for quantification of the carbohydrate content in previous studies, de Lima et al., 2018 [81], reported that application of a wavelength range from 908 to 1676 nm was sufficient to conduct a 100% successful two-group classification, i.e., between lactose-free milks and regular milks.

In cheeses, the prediction of macro-component concentrations was similar to those in milk, with the fat and moisture contents being sufficiently predicted but the protein content remaining challenging. For instance, Ma et al., 2019 [82], applied a wavelength range of 740 to 1070 nm for cheese protein assessments, but intact casein (R^2^_P_ 0.61–0.70, RMSEP 0.91–1.58 g/100 g) and total protein content (R^2^_P_ 0.54–0.62, RMSEP 0.62–0.88 g/100 g) were only approximately predicted. 

The discrimination of farming regimes, particularly dairy products from organic and nonorganic production systems, was reported to be classified correctly with a 73–89% accuracy in retail milk samples [83]. Furthermore, Behkami et al., 2019 [84], reported a 100% correct classification rate in regard to the geographical origin of freeze-dried milk applying a range from 200–2600 nm. 

Substitution of milk products to boost the protein content measured by wet-chemical methods or the addition of cheap ingredients to increase volume and weight has occurred mainly in milk powders and infant foods in the past. Milk powder substituted with melamine, dicyandiamide, aminotriazole, biuret, cyanuric acid as low-molecular-weight, nitrogen-rich compounds, inorganic salts (ammonium sulfate and calcium carbonate), soy protein isolate, pea protein isolate, maltodextrin, and sucrose was analysed by Karunathilaka et al., 2018 [85]. In line with the low prediction rates of protein contents in the aforementioned studies, specificity was low, and only biuret-spiked samples were sufficiently identified above 0.4% (*w/w*) biuret in blends. Another unapproved enhancement of milk powders is reported to be the treatment with gamma-irradiation to improve the microbial quality illicitly. Kong et al., 2013 [86], studied milk powder samples with doses of 0, 1.5, 3.0, 4.5, and 6.0 kGy irradiated with 60Co γ-rays using a dose rate of 2 kGy/h that were subjected to NIR measurements in the range of 325–1075 nm. Best prediction results were achieved by application of selecting an effective wavelength and extreme learning machine models (R^2^_P_ 0.97 and RMSEP of 0.844).

Overcoming the limitations of NIR devices on compositional prediction of dairy products, a portable device that scans the MIR region between 4000 and 650 cm^−1^ was successfully applied to predict protein, fat, and carbohydrate contents with values for R^2^_P_ of 0.96, 0.69, and 0.92, respectively, and for RMSECV of 0.22, 0.63, and 0.40 g macronutrients/100 mL, respectively [25]. In the same range, Limm et al., 2018 [23], reported a successful classification of melamine-adulterated milk powders with a 100% correct classification rate for wet-blended and dry-blended mixtures above 0.3% (*w*/*w*) and 1.0% (*w*/*w*), respectively. Milk powders were spiked with melamine from 3% to 10% (*w*/*w*) and analysed with Raman technology recording 200 to 2000 cm^−1^. Results showed a good prediction with an R^2^ of 0.995 and an RMSECV of 33.60 [87]. 

One disadvantage of LIBS is the challenging measurement of liquid samples. According to Sezer et al., 2018 [88], the interaction of the laser beam and the liquids leads to splashing on the detector node as well as aerosol and ripple formation of the sample. Overcoming these issues, Moncayo et al., 2017 [89], used freeze-dried and pelleted milk. With LIBS technology, different origins of milk, i.e., cow, goat, and sheep as well as mixtures thereof, were classified 100% correctly. Furthermore, the addition of melamine to milk was quantified at a high accuracy (R^2^ 0.999). Another option for preprocessing was reported by Sezer et al., 2018 [88], using the Applied Spectra five-channel Aurora LIBS spectrometer. The authors prepared gel samples using gelatin and successfully identified adulterations of caprine and ovine milk blended in bovine milk (caprine R^2^_v_ 0.993, RMSEP 3.56; ovine R^2^_v_ 0.995, RMSEP 4.53). The same device was also used to detect butter adulterated with margarine from 5% to 50% (*w*/*w*), leading to R^2^_v_ of 0.984 and RMSEP of 3.37. Bilge et al., 2016 [90], analysed whey adulteration of milk powder from 1% to 40% (*w*/*w*) by LIBS. The authors reported high R^2^_v_ values of 0.981 for sweet whey and 0.985 for acid whey, indicating a successful detection of whey adulteration in milk powder. 

In brief, the prediction of milk and milk products’ components as well as the substitution or dilution with fraudulent substances is challenging for VIS and NIR devices. In contrast, devices applying MIR, Raman spectroscopy, or LIBS technology are more competent to predict the macro composition and classify correctly. The latter, however, needs for liquid dairy samples an additional pretreatment because liquids are not suitable for this type of measurement.

#### 3.1.3. Fish and Seafood

Two different NIR devices have been used for the authentication of fish (Supplementary Material—Table S4). Grassi et al., 2018 [91], found a 100% correct classification rate for both fillets and patties using a handheld NIR (950–1650 nm) to discriminate Atlantic cod and haddock. Accordingly, O’Brien et al., 2013 [92], reported a successful classification of fish species (cod/winter cod, mullet/red mullet, and samlet/salmon trout) on whole fish and fillets using a similar instrument. Imaging was used to evaluate fish authenticity via the key factor moisture content. He et al., 2013 [93], reported a successful determination of the moisture content of salmon using two HSI systems with spectral ranges of 400–1000 nm and 897 to 1753 nm, respectively. Both devices delivered information that was valuable for the classification, resulting in R^2^_P_ 0.893, RMSEP 1.513% for the 400–1000 nm range and R^2^_P_ 0.902, RMSEP 1.450% for the 897–1753 range. 

Concerning fish and seafood authenticity assessments, wavelength ranges in the NIR range have been mostly applied, both for single point measurements and for imaging. In addition, sensor arrays, such as electronic noses, might be useful to detect authenticity traits, particularly those related to freshness, in samples that are thick or covered with skin.

#### 3.1.4. Other Food Products of Animal Origin

Other food products of animal origin received little attention (Supplementary Material—Table S5). One study on the freshness of eggs (storage time) was reported using NIR [94]. Honey is the other product of animal origin that received some attention. Using a range between 950 and 1630 nm, Kaszab et al., 2017 [95], were able to predict 100% of the floral origin of honeys, i.e., linden, acacia, polyfloral, and chestnut honey. Similarly, the geographical origin could be predicted with a success rate of 96%. Moreover, Lastra-Mejías et al., 2020 [96], and Stefas et al., 2020 [97], discriminated the nectar and geographical origin of honey. The authors reported accuracies between 96% and 100% using LIBS technology. In addition, Lastra-Mejías et al., 2020 [96], were able to correctly detect adulteration of honey with rice syrup with a success rate of up to 96%.

### 3.2. Food Products of Plant Origin

#### 3.2.1. Fresh and Dried Food Products of Plant Origin

Depending on the fresh fruit appearance, i.e., peel thickness, transparency of the edible parts, and fruit volume, or the homogeneity of the plant-based product, the abilities of portable devices to assess their authenticity (listed in Supplementary Material—Table S6) were more or less successful. Whereas VIS and fluorescence technology devices are known to not penetrate the skin of fruits, NIR wavelength ranges allow measurements of parts of the tissue below the fruit or vegetable skin. Hence, only limited reports on the application of a handheld device in the VIS range (380–700 nm) and fluorescence were found. Vincent et al., 2018 [98], reported a 93% accuracy in discriminating different varieties of apples. Despite this successful application, the follow-up approach with the same samples and device differentiating organic and nonorganic apples was not successful. Recording a similar wavelength range but with monochromatic light inducing fluorescence, Dong et al., 2014 [99], were able to classify teas according to the type of cultivar and type of processing (green or black tea).

Applications of VIS-NIR and NIR devices have been more frequently used for authenticity assessments than has only VIS based technology since they are able to determine the macro composition, i.e., water, protein, and fat contents, in addition to secondary metabolites. In the wavelength range 450–1000 nm, You et al., 2017 [100], were able to discriminate 100% of powders from various plant sources with differing macro composition, such as wheat, bean, corn, rice, and potato in addition to salt and sugar. Similarly, Toivonen et al., 2017 [101], authenticated cherry breeds with differing dry matter content with a device using 285 to 1200 nm (R^2^_P_ > 0.9, RMSEP < 0.74%). Fruits with a similar macro composition but with differing secondary metabolite composition, such as bananas, were also authenticated using a VIS-NIR device in the 367–2388 nm range (carotenoid concentration in pulp: R^2^_P_ 0.96; RMSEP 28.70 nmol/g dry weight). Thereby, β-carotene content was best predicted and—in descending order of accuracy—also α-carotene, c-carotene, and lutein contents [102]. Moreover, with VIS-NIR devices, Ikeogu et al., 2017 [103], successfully authenticated cassava roots according to their total carotenoids (R^2^_P_ 0.88) and dry matter (R^2^_P_ 0.80) content, and Szuvandzsiev et al., 2014 [104], discriminated tomato breeds with differing lycopene contents (R^2^_CV_ 0.75, RMSECV 7.63 mg/100 g), soluble solids content (R^2^_CV_ 0.77, RMSECV 0.51 °Brix), and polyphenol contents (R^2^_CV_ 0.72, RMSECV 1.99 mg/100 g). Further applications of VIS-NIR devices in the authenticity assessment of fresh and dried plant-based products include the detection of wheat flour added to unripe banana flour (0–800 g kg^−1^). For instance, Ndlovu et al., 2019 [105], detected this type of adulteration in high precision (R^2^_P_ 0.99, RMSEP 1.99 g kg^−1^) with a VIS-NIR device measuring from 447 to 1005 nm. Similarly, Rukundo and Danao, 2020 [106], used a handheld VIS-NIR device (780–2500 nm) to successfully detect turmeric powder adulterated with the same product from 0 to 30%. Sugarcane was analysed in the range between 300 and 1100 nm to distinguish grades [107]. The authors reported an 83% correct classification rate of premium grades (Brix-oriented). A good correlation with the mineral content (R^2^_P_ 0.78–0.93, RMSEP 0.57–27.30 mg 100 mL^−1^) was also reported by [108]. This content was used to identify growing regimes. Similarly, Jamshidi et al., 2016 [109], classified fresh products according to their farming regime. In a range between 450 and 1000 nm, the authors reported a 92% correct prediction rate of all samples, while 100% of the unsafe samples were correctly identified. Guidetti et al., 2010 [110], determined differences in the VIS-NIR spectra of grapes from different origin.

Food authentication applications have also been described for NIR devices applying wavelength ranges above 700 nm. Tea varieties were successfully discriminated according to their catechin and caffeine content (R^2^_P_ > 0.91, RMSEP < 10.64 mg/g, [111]), bean breeds according to their protein, starch, and amylose content (R^2^_CV_ > 0.91, [112]), plum varieties in relation to soluble solids content (R^2^_CV_ 0.57, [113]), and sugar beets in regard to their sucrose contents (R^2^_P_ > 0.75, [114]). Furthermore, peanuts were discriminated in valuable high-oleic-acid peanuts and regular peanuts with an up to 100% correct classification rate using a portable NIR [115]. When discriminating different varieties, 84% of fengdou from *Dendrobium officinale Kimura et Migo* (DOK) was correctly predicted in comparison to fengdou from another variety by applying a wavelength range from 908 to 1676 nm [116]. Assessing the feasibility of adulteration detection with handheld NIRs, blended Arabica coffee with peels/sticks or corn, in the concentration range from 1 to 100% (*w*/*w*), was analysed by Correia et al., 2018 [117], using a microNIR device in the wavelength range 908–1676 nm. While a good prediction was registered for the detection of corn adulteration (R^2^_P_ 0.98, RMSEP 4.0%), the detection of peels/sticks was less successful (R^2^_P_ 0.86, RMSEP 11.4%), possibly due to the greater similarity of the adulterant and the authentic sample. The authors further studied the detection of roasting levels in mixtures of Arabica and Robusta coffee, and found good prediction results (R^2^_P_ > 0.96, RMSEP < 6.6%, [117]). Oliveira et al., 2020 [118], mixed three types of paprika powder with potato starch and acacia gum to boost volume and annatto to intensify the colour. Using a DLPR NIRscanTM Nano device (wavelength range 900–1700 nm), they were able to classify over 90% of the samples correctly (R^2^_P_ 0.87–0.97, RMSEP 1.68–2.12%). In the same wavelength range but with another NIR device, turmeric adulteration with metanil yellow (1–25%) (*w*/*w*) was detected with high accuracy (R^2^_P_ > 0.96, RMSEP < 0.89%, [119]). Adulterated oregano with cistus, myrtle, olive leaf, and sumac was correctly classified by up to 98%, in addition to 93% of the authentic oregano classifications, using a miniaturised NIR device [120]. In the wavelength range from 1600 to 2400 nm, spiked soy bean samples with melamine (0.25–2% (*w*/*w*)) could be predicted in a high precision with R^2^ values from 0.94 to 0.99 and RMSEP values around 0.08–0.22% [121]. Of bell pepper samples from different growing regimes, such as those grown outdoors or in greenhouses, 88–91% were correctly classified using another NIR device (1600–2400 nm, [122]). For detecting organic and nonorganic growing regimes, NIR devices were applied successfully on tomatoes [123] and apples (accuracy 96–98%, [124]). To identify growing regimes with fraudulent fertilizer use, the nitrogen content in olive leaves (accuracy 83%, [125]) and spinach leaves (correct classification rate 75–80%), [126] NIR devices were successfully applied. Some fresh and dried plant-based products were also classified according to their geographical origin using NIR devices. Teye et al., 2019 [127], reported an over 90% correct classification rate of rice of different quality grades, countries of origin, and imported versus local production by applying a portable device in the wavelength range of 740–950 nm. Similarly, Zhu and Tian, 2018 [128], identified the geographical origin of apples with an NIR device (900–1700 nm).

By applying MIR wavelength ranges, Manfredi et al., 2018 [129], reported an accuracy of 98% in the classification of different hazelnut cultivars.

Using a handheld Raman device, Guzman et al., 2012 [130], correctly classified 97–100% of olive fruits by their harvesting manner, e.g., picked from trees and collected from the ground. Krimmer et al., 2019 [131], showed that a handheld Raman device could be used to correctly classify 89–98% of maize kernel according to variety and estimation of nutrient content, i.e., carbohydrates, carotenoids, fibres (lignin), and protein. In a study with limited samples, Vargas Jentzsch et al., 2016 [132], showed that Raman devices may be used to detect counterfeit stevia products.

Perez-Rodriguez et al., 2019 [133], and Yang et al., 2018 [134], used portable LIBS devices to classify rice according to its geographical origin with a 84% and 99% accuracy, respectively, for the two instruments. Moreover, coffee adulterated with wheat, corn, and chickpeas was successfully discriminated from authentic coffee using LIBS technology (R^2^_P_ 0.99, RMSEP 6.68–7.85%, [135])

Nutmeg adulterated with 5–60% low-quality plant parts was subjected to HSI, and adulteration was predicted with high accuracy (R^2^_P_ > 0.91). Similarly, Su and Sun, 2017 [136], classified adulterated organic wheat flour with nonorganic ones as well as adulteration with flours from other plants fairly correctly (R^2^_P_ value > 0.97, RMSEP < 0.038) using HSI in a higher wavelength range. 

In brief, the application of optical handheld devices is highly dependent on the plant products’ physical appearance. While dried and ground products may be assessed by multiple devices, fresh products are challenging. A thick skin, e.g., as occurring with avocados or oranges, as well as inhomogeneities in the products’ themselves hinder successful authentication. However, these inhomogeneities enable the discrimination of parts of plant-based products, as shown for sugar cane. To overcome homogeneity issues, a wider range has to be tested, either combining multiple single spot measurements or using HSI. Use of LIBS technology is limited since it can only be applied to dried products.

#### 3.2.2. Processed Food Products of Plant Origin

Various applications aiming at processed foods are listed in Supplementary Material—Table S7. Determination of the constitution of syrups and juices is important to the juice industry to avoid food fraud. Henn et al., 2016 [137], showed that a handheld NIR was able to sufficiently quantitate glucose, fructose, and sucrose in syrups (R^2^ > 0.96, RMSECV < 1.84 g/100 g). Adulteration of lime juice leading to unbalanced citric acid-to-iso-citric acid ratios could be detected with 100% accuracy using a portable NIR device [138]. Moreover, adulterations of tabletop sweeteners (saccharin and cyclamate) with sodium saccharin, dextrose, cream of tartar, and calcium silicate (R^2^_P_ 0.97, RMSEP 0.51%), and with sodium cyclamate, dextrose and silicon dioxide (R^2^_P_ 0.96, RMSEP 2.8%), respectively, were sufficiently detected using a NIR device [139]. 

The adulteration of edible oils is a common food fraud issue too, which may be tackled with portable devices. For example, palm oil adulterated with lard from 0.5% to 50% (*w*/*w*) was detected with an accuracy in the range of 0.93% to 0.95% [140] and the adulteration with Sudan dyes (0.5–0.0009%) with a 91% to 95% correct identification rate [141] using a wavelength range from 900 to 1700 nm. Applying the same wavelength range, Yan et al., 2019 [142], were able to successfully discriminate extra virgin olive oil from olive oils of lower quality (94–100% accuracy) and align most components with respective spectral bands. Moreover, the classification in quality grades according to acid value was successful for peanut oil (R^2^_P_ 0.94, RMSEP 0.30, [143]), and palm oil (R^2^_P_ 0.97, RMSEP 4.6, [144]). In addition, the dilution with lower-quality oils and the dilution with toxic, nonedible mineral oils in concentration ranges of 0.5–10% were sufficiently determined (R^2^_CV_ 0.99, RMSEP 0.23–0.32%) according to the studies of Picouet et al., 2018 [145]. Giovenzana et al., 2014 [146], showed that portable NIR devices were able to evaluate beer quality (soluble solid content and acidity) and also allowed discrimination between filtered and unfiltered beer types.

Using portable MIR technology, 100% of edible oils from various sources were discriminated from each other [24,147,148]. In addition, the detection of adulteration above 10% edible oil in olive oil (*w*/*w*) [24] and the evaluation of oil according to its oxidative status and fatty acid composition were successful (R^2^_V_ > 0.96, [147,148]). 

Bellou et al., 2020 [149], evaluated portable LIBS device settings according to their possibilities to discriminate olive oil from different origins and adulterated samples. Thereby, the classification of olive oil being sprayed delivered a 100% correct classification rate, whereas laminar flow and free surface configurations provided less accurate results. To avoid splattering using LIBS, Moncayo et al., 2016 [150], prepared gel samples from wines and Tian et al., 2017 [151], dried wine drops before classifying up to 100% of the samples according to their geographical origins correctly. 

Vargas Jentzsch and Ciobotă, 2014 [152], used a handheld Raman device to discriminate up to 100% of samples correctly according to their variety. Using another Raman handheld device, Zou et al., 2009 [153], reported a distinguishing ability between pure and adulterated olive oils samples above 5% (*v*/*v*).

## 4. Handheld Applications: Type of Commodity versus Technology

Various technologies have been implemented in very small and handy devices that are attractive to users. The applied type of technology and wavelength range vary across applications. An overview of the number of handheld applications according to type of technology and food commodity reviewed in this paper is provided in Table 2. 

More applications focus on products of plant origin (92 out of 150) than on products of animal origin (58 out of 150). This is somewhat surprising considering that when food fraud databases are examined, animal products show higher incidence rates than plant-based products do. Furthermore, animal-based product supply chains are in general considerably more vulnerable to fraud than their plant-based counterparts are [154] and would, therefore, require more mitigation measures. Over three quarters of the applications for products of plant origin focus on fresh/dried products. Even those aiming at “processed products” consider relatively simple processed foods, such as juices and oils. Although the number of applications has grown in the past years, it is often already quite complex to authenticate a single component product (e.g., ground beef or a fish fillet) or a food product after limited processing (e.g., a vegetable oil). Even with laboratory-based technology, authentication of composite and/or highly processed food products is still in its infancy. Among the products of animal origin, meat (products) received most attention, followed by milk and milk products. The technology of choice for handheld applications marginally differs across product groups. NIR devices appear the most popular technology across the board.

Most applications used NIR (80 out of 150), followed by VIS-NIR (23 out of 150) and LIBS (14 out of 150). For the other types of technology, such as fluorescence spectroscopy, MIR, Raman spectroscopy, LIBS, imaging, and sensory arrays, a few applications (2–8) have been reported. Spectra generated by the technologies are in some cases indirectly used for authentication purposes. In this approach, spectral data are calibrated against reference methods in order to estimate concentrations of specific compounds or the extent of other types of features (e.g., colour). The compounds/features’ numbers are subsequently used for authentication purposes. This approach mimics the procedures for, e.g., protein content measurements using NIR bench top devices, which is very commonly used across the globe. Spectra are also frequently used for fingerprint methods. In this mode, the spectra are directly used to set spectral specifications for product groups, allowing them to be distinguished using multivariate statistics. NIR applications use both types of approaches. The VIS wavelength range is in most cases not useful on its own to authenticate foods. However, the inclusion of the VIS range in addition to a wide NIR (and/or MIR) wavelength range often increases performance. That is one of the reasons why VIS-NIR applications are also relatively popular. In the VIS-NIR applications group, we also find the two types of approaches mentioned above. The benefit of reflectance spectroscopy is its noninvasive nature, and devices are relatively light and compact. Good performance, if products differ clearly in composition, in combination with the practical handling and reasonable costs, has resulted in a large share in the portable authentication applications. However, sometimes a greater depth of excitation wavelength or penetration through package material or thick peels is necessary. For this kind of application, Raman instruments as well as LIBS are favoured. However, the downside of these technologies is that they are found in more bulky and heavier variants than the ones based on reflectance spectroscopy, and liquids are challenging. Another technology that is not used frequently at the moment is fluorescence spectroscopy. This is because of its limited application: only when fluorescent compounds are at stake, e.g., chlorophyll or coumarin, the technology is useful. Similarly, sensor arrays can be useful, but only for unpacked foods that release volatiles or are soluble. This limits broader use of this kind of technology. Imaging and spatially resolved spectroscopy has seen also only a few handheld applications so far, but would be in particular useful for authentication issues that can be picked up through inhomogeneity. This may be the case with small-particle-sized products, such as ground spices or other powdered products, which may be extended with other powdered materials. 

## 5. Outlook

### 5.1. Issues Inhibiting Progress

As outlined in detail in Section 3 and Section 4, there is no single universally applicable technology to assess food authenticity for every food commodity. Adding up to this problem is that for each product and handheld device, a separate (spectral) database needs to be established, making broad implementation of handhelds challenging and potentially expensive. Furthermore, general performance criteria for fingerprint methods are not readily available, they are still under development and discussed in various standardisation groups. Even after the development of a single handheld method for a single product following a defined set of performance criteria, challenges remain for the widespread implementation of the method. The main reason for this is that the handheld methods described in this work rely on predefined databases populated with data from authentic samples. The specific sensor type, brand, or principle and its machine learning statistical procedure to relate unknown sample data to the reference database are not flexible and often not transferrable to other devices or slightly different products. 

The rigidity of the specific handheld type, the spectral reference database, and its machine learning protocol is especially applicable to vibrational spectroscopy handhelds or imaging sensors, spanning the wavelength range from VIS to NIR (400–2500 nm). These handhelds deliver spectra with a relatively low information load, compared to their more technically advanced spectroscopy counterparts, MIR, FTIR, and Raman spectroscopy. This means that spectral databases are not directly transferrable to other VIS-NIR hardware types. Specifications in regard to direct comparison of spectra recorded by different devices are lacking as well as for the demonstration of statistical equivalence of the spectra. Consequently, large resources are required for building and maintaining spectral databases. Unlike direct methods, such as mass spectrometry, for every single product and sensor application, this exercise needs to be repeated.

### 5.2. Potential Solutions

An important potential mitigation measure to the above- mentioned issue is innovative data solutions, which could use spectral data published in open-access databases and use data acquired by different types of vibrational spectroscopic devices. Large volumes of spectral data in regard to food authentication have been generated and could be made available and re-used. Such data would need to be available according to the FAIR principle: Findable, Accessible, Interoperable, and Re-usable. Furthermore, the availability of sensor data resulting from measurements on certified (authentic) materials can facilitate the training and validation of handheld methods according to set performance criteria. To enlarge the impact of the available FAIR data, new data solutions are being developed and tested for adequate calibration transfer of this data, next to the currently established procedures. So-called “federated” learning procedures such as dynamic feature selection are being developed to handle the intrinsic heterogeneity of the (authentic) food materials and machine errors presented in the FAIR-data-containing learning networks. Furthermore, the application and development of deep learning tools will facilitate calibration transfer between spectral sensors with a larger difference in hardware configuration, as an elaborate perspective showed by Müller-Maatsch et al., 2021 [60].

Another part of the solution may be the development of more “universal” devices, i.e., the hyphenation or fusion of several spectral technologies into a single device. Hence, the whole spectral region of interest is recorded at once, preferably including imaging and other orthogonal detection techniques. One example that has been recently reported is the hyphenation of fluorescence, VIS, NIR, and imaging technology into a single sensor for food authentication. While the NIR sensor provided most of the relevant information to authenticate milk powders [155], the fluorescence sensor was most important in olive oil authentication [156]. 

An additional trend is to expand the number of spectral sensor operators to cope with the complexity of the food authenticity problem. Implementation of sensing solutions in entire chains is therefore to be expected from farm-to-fork, dealing with several intermediate products and sensor operators. The information gathered from the spectral data can be added to other types of information from the chain, such as mass balance data, and used for assurance of the integrity throughout the chain as well as for detection of anomalies. The information may also be used for decision support systems, allowing actors to put mitigation measures efficiently and effectively in place. In the end, the collated and analysed (spectral) data and outcomes will lead to increased transparency of food supply chain networks. 

## Figures and Tables

**Table 1:** Technologies for food authentication available in miniaturised form: variants of optical sensors, imaging sensors, sensor arrays, and NMR devices.

Technology	Details	Feature Measured	Availability	Stage (Prototype/Commercial)	Literature (Original Articles and Reviews)
Optical sensors	Reflectance	Colour (CIE lab)	Handheld	C	[17]
	Reflectance, absorption, fluorescence	VIS spectrum	Handheld	P/C	[18]
	Reflectance, absorption	FLUO spectrum	Handheld	P/C	[18,19]
	Reflectance, absorption	NIR/SWIR spectrum	Handheld	P/C	[12,20]
	Reflectance	FTIR spectrum	Portable	P/C	[21,22]
	Reflectance	MIR spectrum	Portable	P	[21,23]
	Raman scattering	Scattered light	Portable/handheld	C	[13,20,24]
	LIBS	Emitting light from plasma cooling	Portable	P/C	[25]
Imaging	Reflectance, fluorescence	VIS spectrum	Portable	P/C	[18,26,27]
	Reflectance	SWIR spectrum	Portable	P/C	[26,27]
	Scattering	Raman spectrum	Portable	P	[24]
NMR	NMR	NMR spectrum	Portable	P	[28]
Sensor arrays	Electronic Nose	Volatile compounds	Handheld	P/C	[10,29,30,31,32,33]
	Electronic Tongue	Solutes	Portable	P	[30,34,35,36]

VIS = visible spectroscopy; FLUO = fluorescence spectroscopy; NIR = near-IR spectroscopy, SWIR = short-wave IR spectroscopy; FT-IR = Fourier-transformed IR; MIR = mid-IR; NMR = nuclear magnetic resonance spectroscopy; LIBS = Laser-induced breakdown spectroscopy; C = commercial; P = prototype.

**Table 2:** Number of studies on handheld applications for food authentication from Supplementary Material—Tables S2–S7 categorised by food product and technology group.

Technology	Products of Animal Origin				Products of Plant Origin		Total
	Meat, Meat Products, and Offal	Milk and Milk Products	Fish and Seafood	Others	Fresh/Dried	Processed	
VIS	2	1	-	-	2	-	5
FLUO	1	1	-	-	2	-	4
VIS-NIR	4	2	-	-	17	-	23
NIR	17	8	3	2	40	10	80
MIR	-	2	-	-	1	3	6
Raman	1	1	-	-	4	2	8
LIBS	2	4	-	2	3	3	14
Imaging	1	-	3	-	4	-	8
Sensor array	-	-	1	-	1	-	2
Total-1	28	19	7	4	74	18	
Total-2	58				92		150

VIS = visible spectroscopy; FLUO = fluorescence spectroscopy; NIR = near-IR spectroscopy, MIR = mid-IR (MIR), Raman = Raman spectroscopy; LIBS = laser-induced breakdown spectroscopy.

## Supplementary Materials

The following are available online at https://www.mdpi.com/article/10.3390/foods10122901/s1, Table S1: Overview of commercially available, portable devices that may be applied for food authentication, their weights and volumes as presented in Figure 1. Table S2: Applications of commercially available devices that may be applied for authentication of meat, meat products, and offal, Table S3: Applications of commercially available devices that may be applied for authentication of milk and milk products, Table S4: Applications of commercially available devices that may be applied for authentication of fish and seafood, Table S5: Applications of commercially available devices that may be applied for authentication of products of animal origin other than those presented in Tables S2–S4, Table S6: Applications of commercially available devices that may be applied for authentication of fresh and dried food products of plant origin, Table S7: Applications of commercially available devices that may be applied for authentication of processed food products of plant origin. References [157,158,159,160,161,162,163,164,165,166,167,168,169,170,171,172,173,174,175,176,177,178,179,180,181,182,183,184,185,186,187,188,189,190,191,192,193,194,195,196,197,198,199,200,201,202,203,204,205] are cited in the supplementary materials.
