# Supplementary material for: Handheld Devices for Food Authentication and Their Applications: A Review
Source: Foods. 2021 Nov 23;10(12):2901. doi: 10.3390/foods10122901 (PMC8700508; doi:10.3390/foods10122901)
Supplement: Supplementary file 1 [file foods-10-02901-s001.zip › foods-1472209-supplementary.pdf]

Supplementary Materials

Handheld Devices for Food Authentication and Their Applications: A Review

Judith Müller-Maatsch, Saskia van Ruth

Table S1. Overview of commercially available, portable devices that may be applied for food authentication, their weights and volumes as presented in Figure 1. Courtesy to instrument suppliers' websites.

**Technology**	**Brand**	**Type**	**Weight [kg]**	**Volume [cm^3]**
Ultraviolet-visible spectroscopy	ASD	FieldSpec	1.2	2640
Ultraviolet-visible spectroscopy	ASD	QualitySpec	2.5	9300
Ultraviolet-visible spectroscopy	OceanOptics	HR2000 4000	0.6	702
Ultraviolet-visible spectroscopy	OceanOptics	USB2000 4000	0.2	194
Ultraviolet-visible spectroscopy	OceanOptics	FLAME	0.3	180
Ultraviolet-visible spectroscopy	OceanOptics	JAZ	2.2	16335
Ultraviolet-visible spectroscopy	OceanOptics	QE65000 65 pro	1.2	1064
Ultraviolet-visible spectroscopy	FelixIns	F 750	1.1	1023
Ultraviolet-visible spectroscopy	Avantes	AvaSpec	0.2	129
Ultraviolet-visible spectroscopy	Avantes	AvaSpecLIBS	0.2	129
Ultraviolet-visible spectroscopy	Andor Oxford Instruments	Mechelle 5000	10.0	22341
Ultraviolet-visible spectroscopy	Stratio	Linksquare	0.1	14
Ultraviolet-visible spectroscopy	Minolta	CM2002 CR400	1.6	3724
Ultraviolet-visible spectroscopy	Variable Spectros	ColorMuse	<0.1	75
Fluorescence spectroscopy	OceanOptics	USB2000 4000	0.2	194
Infrared spectroscopy	Si-Ware	Neospectra	<0.1	77
Infrared spectroscopy	Spectral Engines	NIRONE	<0.1	11
Infrared spectroscopy	Innospectra	NIRS G1	<0.1	207
Infrared spectroscopy	Consumerphysics	Scio	<0.1	51
Infrared spectroscopy	Thermo Fisher	TruDefender	1.3	1163
Infrared spectroscopy	Viavi	MicroNIR 2200	0.1	131
Infrared spectroscopy	Viavi	MicroNIR	0.1	79
Infrared spectroscopy	TellSpec	Enterprise Sensor	0.1	244
Infrared spectroscopy	ASD	LabSpec 2500	12.0	13647
Infrared spectroscopy	OceanOptics	NIRQuest	1.2	941
Infrared spectroscopy	Texas Instruments	DLPNIRScan	0.1	129
Infrared spectroscopy	Control Development Inc	NIR512L-1.7T1	0.9	1331
Infrared spectroscopy	Agilent	Exoscan FTIR	3.2	4558
Infrared spectroscopy	Agilent	4500 ATR-FTIR	6.8	12122
Infrared spectroscopy	Agilent	Cary 630 FTIR	3.6	6000
Infrared spectroscopy	FANTEC	FQANIR GUN	2.8	5000
Infrared spectroscopy	Arcoptix	FT-NIR	1.7	2304
Infrared spectroscopy	JMAnalytik	TIDAS S700	8.0	15758
Raman spectroscopy	OceanOptics	QE65000 65 pro	1.2	1064

Raman spectroscopy	SciAps	Inspector Raman	2.3	1326
Raman spectroscopy	Rigaku	Progeny	1.6	1792
Raman spectroscopy	Rigaku	Xantus	2.2	244
Raman spectroscopy	Metrohm	Raman Sierra	2.7	4690
Raman spectroscopy	Tornado Spectral	HyperFlux PRO Plus	9.8	13819
Raman spectroscopy	OptoTrace	RamTracer	3.0	3240
Raman spectroscopy	Bruker	BRAVO	1.5	2611
Raman spectroscopy	Agilent	Resolve	2.2	3281
Laser induces breakdown spectroscopy	OceanOptics	HR2000 4000	0.6	702
Laser induces breakdown spectroscopy	Avantes	AvaSpec	0.2	129
Laser induces breakdown spectroscopy	Andor Oxford Instruments	Mechelle 5000	10.0	22341
Imaging	Specim	Spectral camera IQ	1.3	1414
Imaging	Specim	Spectrograph ImSpector	4.5	4550
Imaging	Xenics	XEVA FPA	1.3	1065
Sensor arrays	Cyrano Sciences	Cyranose 320	0.9	1100
Sensor arrays	Airsense	PEN3	2.1	4457
Sensor arrays	FoodSniffer	FoodSniffer	0.2	1080

Table S2. Applications of commercially available devices that may be applied for authentication of meat, meat products, and offal.

Food	Targeted food component (if applicable)	Property (if applicable)	Technology	Wavelength range [nm]	Device name	Literature
Lamb carcasses	-	Feeding regime	VIS	400-700	Minolta CM-2002, Konica Minolta, Chizoda, Tokio, Japan	Dian et al., 2008 [62]
Chicken	-	Contamination	VIS<br>NIR	400-900<br>900-1700	EPP2000-CXR, StellarNet Inc., Tampa, FL, USA<br>EPP2000, StellarNet Inc., Tampa, FL, USA	Chao et al., 2008 [157]
Minced beef	-	Spoilage	FLUO<br>VIS-NIR	307-460<br>346-600<br>410-705	USB 2000 spectrofluorimeter, IDIL Fibres Optiques (Lannion, France)	Ait-Kaddour et al., 2011 [158]
Live pigs, pork carcasses	Fatty acid profile	Feeding regimes	VIS-NIR	350-2500	LabSpecPro A108310, ASD, Boulder, Colorado, USA	Perez-Marin et al., 2009 [63]
Pig subcutaneous fat	Fatty acid profile, iodine value	-	VIS-NIR	350-2500	LabSpec4, ASD, Boulder, CO, USA	Prieto et al., 2018 [64]
Beef carcasses	-	pH, color traits, water holding capacity, shear force	VIS-NIR,<br>NIR	350-1830<br>905-1649	LabSpec2500, ASD, Boulder, CO, USA<br>MicroNIR Pro, Viavi Solutions, Santa Rosa, CA, USA	Savoia et al., 2019 [159]
Salted minced pork	Fat and moisture content	-	NIR	740-1070<br>940-1700	Scio, Consumer Physics, Tel Aviv, Israel<br>SpectralProbe, Polychromix, Wilmington, MA, USA	Kartakoullis et al., 2019 [160]

Sausages	-	Superficial water activity (aw) and moisture content during fermentation process	NIR	920-1800<br>1100-2300<br>830-2500	SpectralProbe, Polychromix, Wilmington, MA, USA<br>Luminar 5030, Brimrose Corp. of America, Baltimore, USA<br>Matrix-F duplex FT-NIR, Bruker Optik GmbH, Ettlingen, Germany	Collell et al., 2012 [161]
Pork muscles	Crude protein, moisture content, fat content	-	NIR	1600-2400	Phazir 1624, Polychromix, Wilmington, MA, USA	Zamora-Rojas et al., 2012 [69]
Pork carcasses	-	Feeding regime	NIR	1600-2400	Phazir 1624, Polychromix, Wilmington, MA, USA	Zamora-Rojas et al., 2012 [69]
Pork carcasses	-	Shelf life (Colony forming units, pH)	NIR	1600-2400	Phazir, Polychromix, Wilmington, MA, USA	Prado et al., 2011 [162]
Pork carcasses	-	Quality classification by breeding (DNA analysis), feeding regimes	NIR	1600-2400	MicroPhazir 1624, Polychromix, Wilmington, MA, USA	Horcada et al., 2020 [70]
Veal sausages	-	Classification of species, Detection of adulteration with pork meat and fat	NIR	1600-2400	MicroPhazir GP4.0 ThermoFisher Scientific, MA, USA (former Polychromix)	Schmutzler et al., 2015 [74]
Pork	-	Freshness (Thiobarbituric acid reactive substances values)	NIR	1100-2200	MicroNIR 2200, Viavi Solutions, San Jose, CA, USA	Kucha & Ngadi 2020 [163]
Pork carcasses	-	Premium classifications according to fatty acid composition	NIR	900-1700	MicroNIR Onsite Lite, Viavi Solutions, Santa Rosa, CA, USA	Piotrowski et al., 2019 [68]

Chicken breast fillet	-	Animal welfare classification, Non-organic/organic, feeding regimes, storage properties (frozen-thawed)	NIR	908-1676	MicroNIR Pro, Viavi Solutions, Milipats, CA, USA	Parastar et al., 2020 [71]
Game muscles	-	Classification of species	NIR	908-1700	MicroNIR OnSite, Viavi Solutions, San Jose, CA, USA	Dumalisile et al., 2020 [73]
Pork carcasses	-	Premium classification according to fatty acid composition	NIR	910-1676	MicroNIR Pro1700, Viavi Solutions, San Jose, CA, USA	Perez-Marin et al., 2021 [67]
Pork, chicken, beef minced meat	-	Classification of species, adulteration detection	NIR	908-1676	MicroNIR Pro1700, Viavi Solutions, San Jose, CA, USA	Silva et al., 2020 [75]
Lamb	Fat content	Age when slaughtered	NIR	900-1700<br>350-2500<br>350-2500<br>350-2500	NIRScan Nano, Texas instruments, Texas, USA<br>LabSpec4, ASD, Boulder, CO, USA<br>LabSpec5000, ASD, Boulder, CO, USA<br>FieldSpec Trek, ASD, Boulder, CO, USA	Dixit et al., 2020 [66]
Chicken parts	-	Discrimination of chicken parts	NIR	900-1700	NIRScan Nano, Texas instruments, Texas, USA	Nolasco Perez et al., 2018 [72]
Chicken breast fillets	-	Spoilage	Raman	784-1135	QE Pro-Raman spectrometer, Ocean Optics, the Netherlands	Jaafreh et al., 2018 [164]

Minced beef	-	Classification of species, adulteration	LIBS	300-900	Aurora LIBS spectrometer Applied Spectra, Fremont, CA, USA	Bilge et al., 2016 [76]
Minced beef	-	Substitution with offal	LIBS	185-904	LIBSCAN 1500, Applied Photonics Limited, Skipton North Yorkshire, United Kingdom	Casado-Gavalda et al., 2017 [77]
Chicken	-	Contamination of chicken	Imaging	430-900	ImSpector V9, Specim, Spectral Imaging, Oulu, Finland	Liu et al., 2003 [165]

VIS = visible spectroscopy; FLUO = fluorescence spectroscopy; NIR = near-IR spectroscopy; MIR = mid-IR, Raman = raman spectroscopy; LIBS = laser induced breakdown spectroscopy; - = not applicable/available.

Table S3. Applications of commercially available devices that may be applied for authentication of milk and milk products.

Food	Targeted food component (if applicable)	Property (if applicable	Technology	Wavelength range [nm]	Device name	Literature
Cheese	-	Feeding regimes	VIS	400-700	Minolta CM-2002, Konica Minolta, Chizoda, Tokio, Japan	Andueza et al., 2013 [78]
Cheese	Fat, dry matter, pH, total and soluble protein	-	FLUO	307-460<br>346-600<br>410-705	Spectrometer,<br>Idil Fibres Optiques, Lannion, France	Karoui & Dufour 2008 [166]
Milk	Fat, protein content	-	VIS-NIR	400-995	Tidas S 700,<br>J &M Analytik AG, Essingen, Germany	Bogomolov et al., 2017 [80]
Milk powder	-	Treatment with gamma irradiation	VIS-NIR	325-1075	FieldSpec<br>ASD, Boulder, CO, USA	Kong et al., 2013 [86]
Milk	Fat, protein content, lactose	-	NIR	1100-1400<br>1700-200<br>2200-2500	Nirone 1.4, Nirone 2.0, Nirone 2.5, Spectral Engines Oy, Helsinki, Finland	Uusitalo et al., 2019 [79]
Cheddar cheese	Total protein content	Ripening stage/ proteolysis (intact casein content)	NIR	740-1070	Scio, Consumer Physics, Tel Aviv, Israel	Ma et al., 2019 [82]
Cheese (various types)	Fat and moisture content	-	NIR	740-1070	Scio, Consumer Physics, Tel Aviv, Israel	Wiedemair et al., 2019 [167]
Milk	Protein, fat and carbohydrate content	-	NIR	740-1070<br>1350-2558	Scio, Consumer Physics, Tel Aviv, Israel<br>Neospectra<br>Si-Ware, Cairo, Egypt	Riu et al., 2020 [168]

Retail milk	-	Organic/non-organic dairy farming	NIR		Micro-NIR 1700, Viavi Solutions, San Jose, CA, USA	Liu et al., 2018 [83]
UHT milk	-	Regular/lactose-free milk	NIR	908-1676	Micro-NIR 1700, Viavi Solutions, San Jose, CA, USA	de Lima et al., 2018 [81]
Milk powder	-	Adulteration	NIR		Phazir Thermo Fisher Scientific, Waltham, MA, USA (former Polychromix)	Karunathilaka et al., 2018 [85]
Freeze-dried milk	-	Geographical origin	NIR	200-2600	Arcoptix FTIR-NIR Arcoptix, Neuchatel, Switzerland	Behkami et al., 2019 [84]
Milk powder	-	Adulteration	MIR	15385-2500	Agilent 4500 portable ATR-FTIR spectrometer, Agilent Technologies, Inc., Santa Clara, CA, USA	Limm et al., 2018 [23]
Milk	Protein, fat carbohydrate content	Qualitative prediction of lactose	MIR	2500-16667	ATR-FT-MIR 4100 Exoscan, Agilent Technologies, Inc., Santa Clara, CA, USA	Gorla et al., 2020 [25]
Milk powder	-	Adulteration	Raman	5000-50000	Inspector RamanTM 1.3, DeltaNu Inc., Laramie, WY, USA	Cheng et al., 2010 [87]
Milk powder	-	Adulteration	LIBS	540-900	HR 4000 , Oceanoptics, Dunedin, FL, USA	Bilge et al., 2016 [90]
Milk (freeze-dried)	-	Species detection, Adulteration	LIBS	190-450	AvaSpec, Avantes, Apeldoorn, the Netherlands	Moncayo et al., 2017 [89]

Milk	-	Species detection	LIBS	186-900	Aurora, Applied Spectra, Fremont, CA, USA	Bilge et al., 2016 [76]
Butter	-	Adulteration	LIBS	186-900	Aurora, Applied Spectra, Fremont, CA, USA	Temiz et al., 2018 [169]

VIS = visible spectroscopy; FLUO = fluorescence spectroscopy; NIR = near-IR spectroscopy; MIR = mid-IR; (ATR)-FTIR = (attenuated total reflectance) Fourier transform IR; Raman = raman spectroscopy; LIBS = laser induced breakdown spectroscopy; - = not applicable/available.

Table S4. Applications of commercially available devices that may be applied for authentication of fish and seafood.

Food	Targeted food component (if applicable)	Property (if applicable)	Technology	Wavelength range [nm]	Device name	Literature
Fish fillets and patties	-	Species detection	NIR	950-1650	MicroNIR OnSite, Viavi Solutions, Santa Rosa, CA, USA	Grassi et al., 2018 [91]
Fish fillets and patties	-	Species detection	NIR	887-1667	MicroNIR 1700, Viavi Solutions, Santa Rosa, CA, USA	O'Brien et al., 2013 [92]
Frozen whole fish	Fat content	-	NIR	600-1000	FT 20, Fantec Research Institute, Kosai, Japan	Shimamoto 2003 [170]
Fish fillets	Moisture content	-	Imaging	400-100<br>897-1753	Specim V10E, Spectral Imaging Ltd., Oulu, Finland<br>ImSpector N17E, Specim, Spectral Imaging, Oulu, Finland	He et al., 2013 [93]
Fish fillets	-	Freshness	Imaging	897-1753	ImSpector N17E, Specim, Spectral Imaging, Oulu, Finland	He et al., 2013 [93]
Shrimp	-	Freshness	Imaging	380-1028	ImSpector V10, Specim, Spectral Imaging, Oulu, Finland	Yu et al., 2018 [171]
Fish fillets	-	Freshness	Sensor array		PEN3, Airsense Analytics, Schwerin, Germany<br>FoodSniffer, Foodsniffer Redwood City, CA, USA	Castrica et al., 2019 [172]

NIR = near-IR spectroscopy; - = not applicable/available.

Table S5. Applications of commercially available devices that may be applied for authentication of products of animal origin other than presented in Tables 2-4.

Food	Targeted food component (if applicable)	Property (if applicable)	Technology	Wavelength range [nm]	Device name	Literature
Eggs	-	Freshness (Storage time)	NIR	740-1070	Scio, Consumer Physics, Tel Aviv, Israel	Coronel Reyes & Ramirez-Morales 2018 [94]
Honey	-	Nectar origin, geographical origin	NIR	950-1630	TellSpec Tellspec Inc., Toronto, Ontario, Canada	Kaszab et al., 2017 [95]
Honey	-	Nectar origin, geographical origin	LIBS	200-1000	AvaSpec, Avantes, Apeldoorn, the Netherlands	Stefas et al., 2020 [97]
Honey	-	Nectar origin, geographical origin, Detection of adulteration with rice sirup	LIBS	200-980	BRC115P–V, B&WTEK, Newark, USA	Lastra-Mejías et al., 2020 [96]

NIR = near-IR spectroscopy; LIBS = laser induced breakdown spectroscopy; - = not applicable/available.

Table S6. Applications of commercially available devices that may be applied for authentication of fresh and dried food products of plant origin

Food	Targeted food component (if applicable)	Property (if applicable)	Technology	Wavelength range [nm]	Device name	Literature
Tomato	Carotenoid content	Freshness/ Storage days	VIS Raman	-	CR-400, Konica Minolta, Chizoda, Tokio, Japan QE Pro-Raman spectrometer, Ocean Optics, Dunedin, FL, USA	Fu et al., 2016 [173]
Apples	-	Classification of variety, organic vs non-organic	VIS	-	SPARKS, Ocean Optics, Dunedin, FL, USA	Vincent et al., 2018 [98]
Tea	-	Classification	FLUO	-	USB 2000, Ocean Optics, Dunedin, FL, USA	Dong et al., 2014 [99]
Maize kernels	-	Contamination with mycotoxins	FLUO	-	AvaSpec 2048, Avantes, Apeldoorn, the Netherlands	Smeesters et al., 2015 [174]
Food powders (plant and non-plant origin)	-	Classification	VIS-NIR	450–1000	Linksquare, Avantes, the Netherlands	You et al., 2017 [100]
Whole melons	Color, total soluble solids	Ripeness (based on the ration of the edible portion)	VIS-NIR		QE Pro-spectrometer, Ocean Optics, the Netherlands	Wang et al., 2014 [44]
Intact oranges	Soluble solids content	Ripeness	VIS-NIR	360-2000	QE Pro-spectrometer, Ocean Optics, the Netherlands	Wang et al., 2014 [44]
Banana fruit pulp	Carotenoid content	-	VIS-NIR		LabSpec2500, ASD, Boulder, CO, USA	Davey et al., 2009 [102]

Cassava roots	Dry matter content, carotenoid content	-	VIS-NIR		QualitySpec, ASD, Boulder, Colorado, USA	Ikeogu et al., 2017 [103]
Turmeric powder	-	Detection of adulteration	VIS-NIR	780-2500	QualitySpec, ASD, Boulder, Colorado, USA	Rukundo & Danao 2020 [106]
Sugarcane pieces	Soluble solids content, fiber content, moisture content	Sample type, quality classes	VIS-NIR	325-1075	Fieldspec, ASD, Boulder, CO, USA	Mat Nawi et al., 2013 [107]; Mat Nawi et al., 2013 [175]
Tomato puree	Soluble solids content, antioxidant components	-	VIS-NIR	500-1000	Fieldspec, ASD, Boulder, CO, USA	Szuvandzsiev et al., 2014 [104]
Intact mango	Soluble solid content	Fruit quality	VIS-NIR	600-1000	FT 20, Fantec Research Institute, Kosai, Japan	Saranwong et al., 2003 [176]
Intact mango	Dry matter, soluble solids content, firmness	Maturity, fruit quality	VIS-NIR	310-11400	F 750, Felix instruments, Washington, USA	Santos Neto et al., 2018 [177], dos Santos Neto et al., 2017 [178]
Unripe banana flour	-	Adulteration	VIS-NIR	447–1005	F 750, Felix instruments, Washington, USA	Ndlovu et al., 2019 [105]
Cherry	Dry matter	Fruit quality	VIS-NIR	285–1200	F 750, Felix instruments, Washington, USA	Toivonen et al., 2017 [101]
Intact orange	Soluble solids content	Fruit quality	VIS-NIR	600-1100	NIRmagic, Beijing Weichuangyingtu Technology, China	Li et al., 2020 [179]

Intact and pressed grapes, blueberries	Soluble solids content, tritratable acidity, pH, polyphenol content, anthocyanin content	Fruit quality/ripeness	VIS-NIR	450-980	AvaSpec 2048, Avantes, Apeldoorn, the Netherlands	Guidetti et al., 2010 [110]
Sugarcane	Mineral content	Fruit quality	VIS-NIR	350-1000	JAZ-EL350, Ocean Optics, Dunedin, FL, USA	Steidle Neto et al., 2017 [108]
Apples	Total soluble solids (Brix), titratable acids, firmness, phenols, anthocyanins, flavonoids, chlorophyll, carotenoids, ascorbate (Vit C)	Nutritional value	VIS-NIR	450-980	AvaSpec 2048, Avantes, Apeldoorn, the Netherlands	Beghi et al., 2012 [180]
Intact oranges	Soluble solids content, acidity (pH), titratable acids, firmness, juice volume, fruit weight, rind weight	Maturity	VIS-NIR<br>NIR	350–1000<br>1100–2300	LabSpec2500, ASD, Boulder, CO, USA<br>Luminar 5030, Brimrose Corp. of America, Baltimore, USA	Cayuela & Weiland 2010 [181]
Fresh fruits (kiwifruits, feijoa fruit, avocado, apples)	Dry matter, total soluble solids, firmness, titratable acids, color	Fruit quality, maturity	NIR	740-950	Scio, Consumer Physics, Tel Aviv, Israel	Li et al., 2018 [182]
Fresh fruits (kiwifruits, apples, apricots, nectarines, peaches, plums)	Dry matter content	-	NIR	740-950<br>740-950<br>650-950<br>600-1000	Scio, Consumer Physics, Tel Aviv, Israel<br>H100C, Sunforest, Incheon, Korea<br>Nirvana, Integrated Spectronics, Baulkham Hills, Australia	Kaur et al., 2017 [183]

					F 750, Felix instruments, Washington, USA	
Rice	-	Authenticity, quality grades, geographical origin	NIR	740-950	Scio, Consumer Physics, Tel Aviv, Israel	Teye et al., 2019 [127]
Buckwheat grains	Antioxidant capacity	Quality	NIR	740-950<br>1596-2396<br>8865-4627	Scio, Consumer Physics, Tel Aviv, Israel<br>MicroPhazir RX, Polychromix, Wilmington, MA, USA<br>MicroNIR 2200, Viavi Solutions, San Jose, CA, USA	Wiedemair & Huck 2018 [184]
Oregano	-	Detection of adulteration	NIR	1350–2500	Neospectra, SI-ware, Heliopois, Egypt	McVey et al., 2021 [120]
Tea leaves	Catechin content, caffeine content	Quality	NIR	900-1700	NIR-S-R2, InnoSpectra, Taiwan	Wang et al., 2020 [111]
Olive fruits	Oil content, genotype	Fruit quality, ripeness	NIR	1100 - 2300	Luminar 5030, Brimrose, Baltimore, USA	León & Gracia 2011 [185]
Olive fruits	Water content, fat content, free acidity	Fruit quality, ripeness	NIR	1100–2300	Luminar 5030, Brimrose, Baltimore, USA	Fernandez-Espinosa 2016 [186]
Olive fruits	Firmness, total chlorophyll, carotenoids,	Ripeness	NIR	1100–2300	Luminar 5030, Brimrose, Baltimore, USA	Cirilli et al., 2016 [187]

	anthocyanins, polyphenols					
Olive leaves	Nitrogen content	-	NIR	1100-2500<br>1100-1700<br>450-1000	Luminar 5030, Brimrose, Baltimore, USA<br>LIGA mini spectrometer, Boehringer-Ingelheim, Ingelheim, Germany<br>USB 2000, Ocean Optics, Dunedin, FL, USA	Rotbart et al., 2013 [125]
Soy	-	Adulteration with melamine	NIR	1600-2399	MicroPhazir, ThermoFisher Scientific, Dublin, Ireland	Haughey et al., 2015 [121]
Bell peppers	Dry matter, soluble solids content	Growing systems (outdoor versus greenhouse)	NIR	1600-2400	MicroPhazir, Polychromix, Wilmington, MA, USA	Sanchez et al., 2019 [122]
Intact mandarins	Fruit weight, axial and equatorial diameter, color, firmness, pericarp thickness, soluble solids content, pH, titratable acidity, juice content	Maturity	NIR	1600 - 2400	Phazir 2400, Polychromix, Wilmington, MA, USA	Sánchez et al., 2013 [188]
Avocado	Moisture content, dry matter	Maturity	NIR	940–1798	Phazir 1018, Polychromix, Wilmington, MA, USA	Blakey 2016 [189]

Intact nectarines	Fruit weight, diameter, soluble solid content, firmness	-	NIR	1600–2400	Phazir 2400, Polychromix, Wilmington, MA, USA	Sánchez et al., 2011 [190]
Intact strawberries	Firmness, soluble solid content, pH, titratable acidity	Fruit quality	NIR	1600 - 2400	Phazir 2400, Polychromix, Wilmington, MA, USA	Sánchez et al., 2012 [191]
Mandarins, Oranges	Fruit weight, equatorial and axial diameter, color, firmness, pericarp thickness, juice weight	-	NIR	1600- 2400	Phazir 2400, Polychromix, Wilmington, MA, USA	Torres et al., 2017 [192]
Spinach	Nitrate content, ascorbic acid content, soluble solid content	-	NIR	1600- 2400	Phazir 2400, Polychromix, Wilmington, MA, USA	Perez-Marin et al., 2019 [126]
Beans	protein content, starch content, amylose	-	NIR	1596-2396	MicroPhazir RX, Polychromix, Wilmington, MA, USA	Plans et al., 2013 [112]
Plums	Firmness, soluble solids content	Fruit quality, ripeness	NIR	1600- 2400	Phazir 2400, Polychromix, Wilmington, MA, USA	Pérez-Marín et al., 2010 [113]
Grapes	Polyphenol content	Fruit quality classification	NIR	908 - 1676	MicroNIR Pro Lite 1700, Viavi Solutions, San Jose, CA, USA	Baca-Bocanegra et al., 2019 [193]
Coffee beans	-	Adulteration	NIR	908-1676	MicroNIR Pro1700, Viavi Solutions, San Jose, CA, USA	Correia et al., 2018 [117]

Mango	Soluble solids content, dry matter, titratable acidity, pulp firmness, soluble solids	Fruit quality	NIR	950–1650	MicroNIR Pro1700, Viavi Solutions, San Jose, CA, USA	Marques et al., 2016 [194]
Peanuts	Fatty acid analysis	Quality classification	NIR	908–1676	MicroNIR Pro1700, Viavi Solutions, San Jose, CA, USA	Yu et al., 2020 [115]
Tomatoes	Fresh weight, pH, dry matter, chromatic values, electrical conductivity, titratable acidity, SSC	-	NIR	908-1650	MicroNIR Pro1700, Viavi Solutions, San Jose, CA, USA	Castrignanò et al., 2019 [123]
Paprika powder	-	Adulteration with colorant and bulk adulterants	NIR	900–1700	DLPR NIRscanTM Nano, Texas instruments, Texas, USA	Oliveira et al., 2020 [118]
Mulberries, Fengdou	Dry matter, soluble solids content, polyphenol content, flavonoid content	Fruit quality, Discrimination of varieties	NIR	908 - 1676<br>909–1649	NIRScan Nano, Texas instruments, Texas, USA<br>MicroNIR Pro1700, Viavi Solutions, San Jose, CA, USA	Yan et al., 2019 [116]
Intact mandarins	Total acids, soluble solids content	-	NIR	350–1040	USB 4000, Ocean optics, Dunedin, FL, USA	Liu et al., 2010 [195]
Cucumbers	-	Farming regime classification, pesticide usage	NIR	450–1000	USB 2000, Ocean optics, Dunedin, FL, USA	Jamshidi et al., 2016 [109]
Intact oranges	Soluble solids content, titratable acids	Fruit quality, maturity	NIR	200-1100	USB 4000, Ocean optics, Dunedin, FL, USA	Liu et al., 2010 [195]

Snake fruit	Soluble solids content	-	NIR	300-1040	USB 4000, Ocean optics, Dunedin, FL, USA	Suhandy et al., 2010 [196]
Apple	Soluble solids content, firmness	-	NIR	900-1700	NIRQuest512, Ocean optics, Dunedin, FL, USA	Zhu & Tian 2018 [128]
Apple	-	Classification in organic and non-organic	NIR	901-1721	NIRQuest512, Ocean optics, Dunedin, FL, USA	Song et al., 2016 [124]
Watermelon	Soluble solid content	Fruit quality, maturity grades	NIR	680–950	FLAME, Ocean Optics, Dunedin, FL, USA	Jie et al., 2013 [197]; Jie et al., 2014 [198]; Jie et al., 2019 [199]
Lambs lettuce	-	Freshness/Decay	NIR	400-1000	JAZ, Ocean Optics, Dunedin, FL, USA	Beghi et al., 2014 [200]
Sugar beet	Mechanical properties, soluble solids content, moisture, sucrose content	Fruit quality	NIR	400–1100<br>900–1600	LOE-USB, tec5USA, Plainview, NY, USA<br>NIR 512L-1.7T1, Control Development, South Bend, IN, USA	Pan et al., 2015 [114]
Tomatoes	Soluble solids content (Brix), pH	Fruit maturity	NIR	400-1100<br>900-1683	LOE-USB, tec5USA, Plainview, NY, USA<br>NIR 512L-1.7T1, Control Development, South Bend, IN, USA	Huang et al., 2018 [201]
Turmeric powder	-	Adulteration with synthetic colorant	NIR	900-1700	Dwarf Star, StellarNet Inc., Tampa, FL, USA	Kar et al., 2017 [119]
Peppercorn	Moisture and phenolic content, antioxidant capacity	-	NIR<br>Imaging	1350-1650<br>400-1000/880-1720	NIROne, Spectral Engines Oy, Finland<br>Spectrograph, Spectral Imaging, Oulu, finland	Esquerre et al., 2020 [202]

Hazelnut	-	Cultivar and geographic origin discrimination	MIR	15385-2500	Exoscan FTIR, Agilent Technologies, Santa Clara, CA, USA	Manfredi et al., 2018 [129]
Olive fruits	-	Harvesting manner	Raman	3704-50000	R3000, Ocean Optics, Dunedin, FL, USA	Guzman et al., 2012 [130]
Maize kernel	-	Nutrient content, variety classification	Raman	5882-25000	Resolve handheld Raman analyser, Agilent Technologies, Santa Clara, CA, USA	Krimmer et al., 2019 [131]
Stevia	-	Counterfeit detection	Raman	5555-25000	Progeny, Rigaku Raman Technologies, Wilmington, MA, USA	Vargas Jentzsch et al. 2016 [132]
Brown rice	-	Geographical origin	LIBS	200-630	HR 4000+ , Oceanoptics, Dunedin, FL, USA	Perez-Rodriguez et al., 2019 [133]
Rice	-	Geographical origin	LIBS	200-950	Mechelle 5000, Oxford Instruments, Tubney Woods, Abingdon, UK	Yang et al., 2018 [134]
Coffee	-	Adulteration	LIBS	186-900	Aurora LIBS spectrometer Applied Spectra, Fremont, CA, USA	Sezer et al., 2018 [135]
Nutmeg	-	Adulteration	Imaging	400-1000	Specim IQ camera, Specim, Spectral Imaging Ltd., Oulu, Finland	Kiani et al., 2019 [203]
Wheat flour	-	Adulteration, Discrimination of	Imaging	900-1700	ImSpector N17E,	Su & Sun 2017 [136]

		organic and non-organic			Specim, Spectral Imaging Ltd., Oulu, Finland	
Citrus	-	Canker detection	Imaging	400-900	ImSpector V10E, Specim, Spectral Imaging Ltd., Oulu, Finland	Qin et al., 2008 [204]
Apple	-	Maturity	Sensor array	-	Cyranose 320, Cyrano Sciences, Pasadena, CA, USA	Pathange et al., 2006 [205]

VIS = visible spectroscopy; FLUO = fluorescence spectroscopy; NIR = near-IR spectroscopy; MIR = mid-IR (MIR), Raman = raman spectroscopy; LIBS = laser induced breakdown spectroscopy; - = not applicable/available.

Table S7. Applications of commercially available devices that may be applied for authentication of processed food products of plant origin.

Food	Targeted food component (if applicable)	Property (if applicable)	Technology	Wavelength range [nm]	Device name	Literature
Sugar syrups	Glucose, fructose, sucrose content	-	NIR	1596-2400	MicroPhazir GP 4.0, Thermo Fisher Scientific, Waltham, MA, USA	Henn et al., 2016 [137]
Tabletop sweeteners	-	Adulteration	NIR	1596-2400	Phazir, Thermo Fisher Scientific, Waltham, MA, USA	Karunathilaka et al., 2017 [139]
Palm oil	-	Adulteration	NIR	908-1650	MicroNIR, Viavi solutions, San Jose, CA, USA	Basri et al., 2017 [140]
Olive oil	Fatty acid composition, chlorophyll and carotenoid concentration, color, moisture	Classification of oils	NIR	908-1676	NIR Pro 1700ES, JDSU, Milpitas, CA, USA	Yan et al., 2019 [142]
Lime juice		Adulteration	NIR	900-1700	TellSpec, Tellspec, Toronto, Canada	Jahani et al., 2020 [138]
Peanut oil	Acid value	Classification of oils	NIR	900-1700	NIRQuest, Oceanoptics, Dunedin, FL, USA	Yang et al., 2017 [143]
Palm oil	Free fatty acids concentration	Classification of oils	NIR	900-1700	TIDA-00554 DLP, Texas Instruments, Dallas, TX, USA	Kaufmann et al., 2019 [144]
Palm oil	-	Adulteration	NIR	900-1700	Visum Palm, Iris, Cornellà de LLobregat Spain	Teye et al., 2019 [141]
Sunflower oil	-	Contamination, adulteration	NIR	833 - 2300	Matrix F Bruker, Ettlingen, Germany	Picouet et al., 2018 [145]
Craft beer	Soluble solids content, pH,	Discrimination of beers	NIR	450-980	AvaSpec2048, Avantes, the Netherlands	Giovenzana et al., 2014 [146]

Olive oil	-	Adulteration, variety discrimination	MIR	4000-650 cm-1	TrueDefender, ThermoScientific, Wilmington, MA, USA	Pan et al., 2018 [24]
Vegetable oils	Peroxide value, fatty acid composition, free fatty acids	Variety discrimination, oil quality (oxidation)	MIR	4000-650 cm-1	TrueDefender, ThermoScientific, Wilmington, MA, USA	Allendorf et al., 2011 [147]
Sacha inchi oils	Peroxide value, fatty acid composition, free fatty acids	Variety discrimination, adulteration	MIR	4000-700 cm-1	TrueDefender, ThermoScientific, Wilmington, MA, USA	Maurer et al., 2012 [148]
Vegetable oils	-	Variety classification	Raman	5000-25000	Xantus, Rigaku Raman Technologies, Wilmington, MA, USA	Vargas Jentzsch & Ciobotă 2014 [152]
Olive oil	-	Adulteration	Raman	4167-41667	RamTracer, OptoTrace Technologies, Inc., Sunnyvale, CA, USA	Zou et al., 2009 [153]
Red wine	-	Geographical origin classification	LIBS	200-1000	EPP2000, StellarNet, Tampa, FL, USA	Moncayo et al., 2016 [150]
Olive oil	-	Geographical origin, adulteration	LIBS	200-1100	AvaSpec-2048-USB2, Avantes, the Netherlands	Bellou et al., 2020 [149]
Red wine	-	Geographical origin classification	LIBS	230-850	Mechelle, Oxford Instruments, Tubney Woods, Abingdon, UK	Tian et al., 2017 [151]

NIR = near-IR spectroscopy; MIR = mid-IR (MIR), Raman = raman spectroscopy; LIBS = laser induced breakdown spectroscopy; - = not applicable/available.
